# The Prevalence, Awareness, and Associated Risk Factors of Inguinal Hernia Among the Adult Population in Saudi Arabia

**DOI:** 10.7759/cureus.65570

**Published:** 2024-07-28

**Authors:** Abdullah Almunifi, Osama A Alshamrani, Shahd M AlMehrij, Abdullah F Alsamhan, Abdulrahman M Althewaikh, Abdullah S Alowaysi, Hussain O Zahid, Saud Aldeghaither, Elsadig Y Mohamed

**Affiliations:** 1 Department of Surgery, College of Medicine, Majmaah University, Majmaah, SAU; 2 College of Medicine, Imam Abdulrahman Bin Faisal University, Dammam, SAU; 3 Department of Surgery, Prince Mohammed Bin Abdulaziz Hospital, Riyadh, SAU; 4 Department of Basic Medical Sciences, College of Medicine, Majmaah University, Majmaah, SAU

**Keywords:** saudi arabia, risks factors, prevalence, inguinal hernia, awareness

## Abstract

Purpose: Inguinal hernias lead to several potentially fatal complications such as strangulation. Assessing the prevalence, risk factors, and beliefs of a population is essential to develop appropriate preventive strategies. This study investigated the prevalence, risk factors, and awareness of inguinal hernia in the adult population of Saudi Arabia.

Methods: This cross-sectional study enrolled 461 adults aged between 18 and 60 years after excluding those aged <18 and >60 years. This study was conducted in five regions of Saudi Arabia (north, west, central, south, and east). A pre-tested questionnaire was used to collect data on the prevalence, perception, and awareness of the participants. Multivariate regression analysis was used to identify risk factors for inguinal hernia.

Results: The study revealed that most participants were men n=262 (56.8%), aged between 18 and 25 years n=241 (52.3%), were single n=278 (60.3%), had a bachelor's degree n=225 (48.8%) and earned less than 50,000 Saudi Riyals annually n=285 (61.8%). Most participants resided in urban areas n=366 (79.4%) with their parents n=230 (49.9%). The prevalence of inguinal hernias in adults was low (5.2%). Our results indicated a significant association between family history of inguinal hernia, chronic cough, bronchial asthma, smoking, and inguinal hernia (p < 0.001). Young adults and undergraduates displayed significantly low awareness of inguinal hernias (p < 0.001).

Conclusion: Family history of inguinal hernia, chronic cough, bronchial asthma, and smoking were factors associated with inguinal hernia. Low awareness levels were observed among young undergraduates, with a moderate overall level of awareness.

## Introduction

Inguinal hernia occurs when an abdominal organ protrudes through the weakened portion of the abdominal wall into the groin; this is a significant public health concern. This protrusion can occur through a defect in the posterior wall of the inguinal canal, resulting in a direct inguinal hernia. Indirect inguinal herniation occurs when a visceral organ projects into the inguinal canal through the internal inguinal ring and exits through it. The organ descends towards the scrotal sac [1,2]. From a clinical perspective, the diagnosis of an inguinal hernia is simple. An essential physical examination can reveal a palpable protrusion or noticeable swelling when pressure is applied using a finger at the external inguinal ring [3].

Inguinal hernia repair is a common surgical procedure [2]. Every year, approximately 500,000 inguinal hernias are treated surgically, with a rate of 217 cases per 100,000 patients undergoing hernia surgery [4,5]. Despite its frequency, inguinal hernia repair may be challenging for even the most experienced surgeons owing to various pathological conditions. Therefore, surgical teams must remain vigilant and well-informed to ensure optimal outcomes [6].

The prevalence of inguinal hernias differs across age groups. The magnitudes of inguinal hernias may vary ranging from as low as 0.06 % [7] to as high as 24.38 % [8]. Approximately 27% of men and 3% of women are affected, with a high incidence observed among the elderly population [9]. According to a recent meta-analysis, the global incidence rate of inguinal hernias was 7.7% [2]. Understanding the overall prevalence of inguinal hernias can emphasize the importance of this condition and help develop appropriate treatment strategies.

Several factors increase the risk of developing an inguinal hernia. Common risk factors include male sex, advanced age, genetic predisposition [10,11], smoking, obesity, persistent coughing, and straining during bowel movements. Thus, it is necessary to identify the risk factors to prevent and treat the condition effectively [10,12,13].

A study among adults in Riyadh, Saudi Arabia revealed a low level of awareness regarding the risk factors associated with hernias. Furthermore, 52% of individuals in Riyadh had a knowledge score below 5 out of 10 concerning risk factors for hernia [14]. In contrast, 38% of adults in Al-Jouf, Saudi Arabia exhibited a high level of knowledge on this subject [15]. However, more research is warranted regarding the general awareness of hernias and their causes, treatment, and prevention among the population in the Kingdom of Saudi Arabia. Thus, this novel study aimed to investigate the prevalence, risk factors, and awareness of inguinal hernia in the adult population of Saudi Arabia.

## Materials and methods

Study design and setting

This cross-sectional study was conducted among adults in Saudi Arabia between March 2023 and February 2024. This study was approved by the Majmaah University Research Ethics Committee (MUREC) (HA-01-R-088), Saudi Arabia (project number is MUREC-MAY.30/COM-2023/20-5). The participants were provided with an explanation of the study's purpose and procedures and gave written informed consent. Patient anonymity was safeguarded and the data were accessible only to the investigators.

Participants

Adult men and women aged between 18 and 60 years living in Saudi Arabia were included in the study, whereas, individuals younger than 18 and older than 60 years were excluded.

Data collection

Data were collected using a pre-tested questionnaire to investigate the prevalence, risk factors, and awareness of inguinal hernia among adults in Saudi Arabia. An online version of the questionnaire was created and distributed equally to the five regions of Saudi Arabia. The questionnaire was divided into two sections: the first part focused on gathering information about the sociodemographic characteristics of the participants, and the second part assessed the risk factors and awareness of inguinal hernia.

Sampling

The sample size was calculated using the following equation: n=(Z^2^×p×q)/d^2^, where n is the population size, Z is the standard average deviation (1.96), p is the prevalence (0.5), q is equal to 1-p (0.5), and d is the accepted error =0.05 [14]. Thus, the sample was taken as 461 participants.

Statistical analyses

The distribution of continuous variables was assessed. Data were presented as medians with interquartile ranges (25th-75th percentiles), and the Shapiro-Wilk test was used to assess the normality of the data. Since the data was not normally distributed, the Chi-square test was used to compare the qualitative data. Categorical variables were expressed as frequencies and percentages and were compared using the Chi-squared test. Multivariate logistic regression analysis was conducted using a stepwise forward selection procedure to identify significant (p < 0.05) risk factors associated with inguinal hernia. These factors were used as candidate variables to determine the risk factors contributing to this condition. Adjusted odds ratios (AOR) were calculated for the coexistence of other predictors and their 95% confidence intervals (CI) to present the obtained results. To calculate the awareness score, a correct answer was assigned a value of 1, whereas an incorrect answer was assigned a value of 0. The level of significance was set at p < 0.05. The Statistical Package for the Social Sciences (SPSS) software program version 26 (IBM Corp., Armonk, NY, USA) was used to analyze the data.

## Results

This study included a total of 461 adults in Saudi Arabia, of which more than half were men n=262 (56.8%). The various age groups included 18-25 n=241 (52.3%), 26-33 n=93 (20.2%), 34-45 n=85 (18.4%), and 46-59 n=42 (9.1%) years. Universities n=225 (48.8%) and secondary schools n=140 (30.4%) education were most common. Approximately two-thirds of n=285 (61.8%) of the study population had an annual income of SR 50,000 or less. Most participants were single n=278 (60.3%) and lived in urban areas n= 366 (79.4%) (Table 1).

**Table 1:** Sociodemographic characteristics (n=461) SR: Saudi Riyal

Characteristics		No.	%
Sex	Female	199	43.2%
	Male	262	56.8%
Age groups, year	18–25	241	52.3%
	26–33	93	20.2%
	34–45	85	18.4%
	46–59	42	9.1%
Marital status	Single	278	60.3%
	Married	150	32.5%
	Divorced	33	7.2%
Education level	Diploma	68	14.8%
	Secondary	140	30.4%
	Bachelor	225	48.8%
	Master degree	19	4.1%
	Doctor of Philosophy	9	2.0%
Annual income/SR	≤50,000	285	61.8%
	51,000–100,000	73	15.8%
	101,000–200,000	67	14.5%
	201,000–500,000	18	3.9%
	More than 500000	18	3.9%
Accommodation	With parents	230	49.9%
	With spouse	141	30.6%
	Alone	59	12.8%
	Student accommodation	16	3.5%
	With a friend	15	3.3%
Residence	Urban	366	79.4%
	Rural	95	20.6%

**Figure 1:**
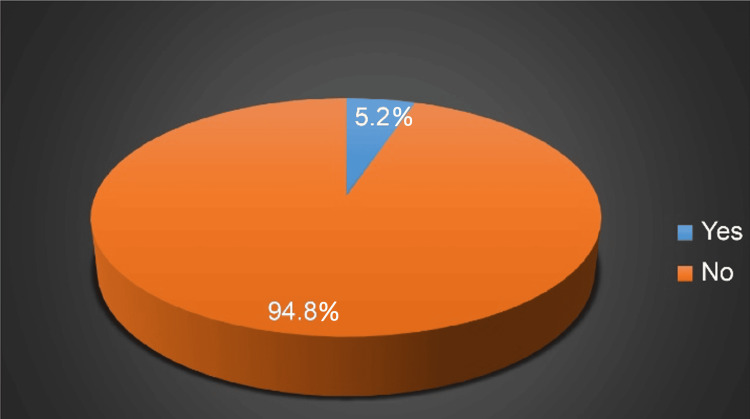
The prevalence of inguinal hernia among the participants

Figure 1 illustrates a 5.2% prevalence of inguinal hernias in the adult population of Saudi Arabia. The significant associations between the presence of an inguinal hernia and a positive family history of inguinal hernia, chronic cough or bronchial asthma, smoking, heavy work or lifting, and chronic constipation or diarrhea (p<0.05) are presented in Table 2. On the contrary, there were no significant associations between inguinal hernia and sex (p=0.318), age (p=0.529), or the presence of benign prostatic hyperplasia (p=0.1000).

**Table 2:** Factors associated with inguinal hernia (n=461) * Significant at p<0.05

Factor		Inguinal hernia				Total		p-Value
		Yes		No				
Sex	Female	8	33.3%	191	43.7%	199	43.2%	0.318
	Male	16	66.7%	246	56.3%	262	56.8%	
Age group, year	18–25	10	41.7%	231	52.9%	241	52.3%	0.529
	26–33	5	20.8%	88	20.1%	93	20.2%	
	34–45	5	20.8%	80	18.3%	85	18.4%	
	46–59	4	16.7%	38	8.7%	42	9.1%	
Family history of inguinal hernia	No	13	54.2%	384	87.9%	397	86.1%	<0.001*
	Yes	11	45.8%	53	12.1%	64	13.9%	
Chronic cough/ bronchial asthma	No	18	75.0%	418	95.7%	436	94.6%	<0.001*
	Yes	6	25.0%	19	4.3%	25	5.4%	
Smoking	No	19	79.2%	424	97.0%	443	96.1%	<0.001*
	Yes	5	20.8%	13	3.0%	18	3.9%	
Obesity	No	19	79.2%	400	91.5%	419	90.9%	0.057
	Yes	5	20.8%	37	8.5%	42	9.1%	
Repeated pregnancy/ hard labor	No	21	87.5%	419	95.9%	440	95.4%	0.089
	Yes	3	12.5%	18	4.1%	21	4.6%	
Heavy work/ lifting heavy things	No	17	70.8%	400	91.5%	417	90.5%	0.004*
	Yes	7	29.2%	37	8.5%	44	9.5%	
Chronic constipation or diarrhea	No	20	83.3%	416	95.2%	436	94.6%	0.034*
	Yes	4	16.7%	21	4.8%	25	5.4%	
Benign prostate enlargement	No	24	100.0%	432	98.9%	456	98.9%	1.000
	Yes	0	0.0%	5	1.1%	5	1.1%	

Multivariable binary logistic regression analysis revealed that a family history of inguinal hernia was significantly associated with an increased risk of inguinal hernia (adjusted odds ratio [AOR]: 6.131, CI: 2.613-14.383, p<0.001). Additionally, individuals with a chronic cough or bronchial asthma were 7.3 times more likely to develop an inguinal hernia, with a significant association (p<0.001). In addition, smoking was significantly associated with an 8.6-fold increased likelihood of inguinal hernia (AOR: 8.583, CI: 2.775-26.548, p<0.001), as shown in Table 3.

**Table 3:** Multivariable binary logistic regression analysis for determining risk factors of inguinal hernia * Significant at p<0.05; AOR: adjusted odds ratio; CI: confidence interval.

	B coefficient	p-value	AOR	95% CI	Accuracy %	p-value
Family history of inguinal hernia	1.813	<0.001*	6.131	2.613–14.383	94.8%	0.001
Chronic cough/bronchial asthma	1.992	<0.001*	7.333	2.613–20.582		
Smoking	2.150	<0.001*	8.583	2.775–26.548		

Table 4 reveals the participants' awareness of inguinal hernias. Approximately two-thirds n=297 (64.4%) recognized the definition of inguinal hernia, n=224 (48.6%) identified the correct location, and n=321 (69.6%) accurately recognized that men over 40, multiparous women, and premature infants were at high risk for inguinal hernia. However, only n=134 (29.1%) gave correct answers regarding those most likely to develop inguinal hernia mandating further clarification. All participants reported that the inguinal hernia manifested as swelling, pain in the inguinal region, or both. Less than two-thirds, n=261 (61.0%), correctly noted that the diagnosis of inguinal hernia depends mainly on physical examination besides radiological examinations and n=140 (30.4%) of participants identified that treatment of inguinal hernia is surgical. Most n=331 (71.8%) respondents identified suggested prevention strategies. The awareness score of the study participants ranged from 1.0 to 8.0, with a median score of 5.0 (IQR: 3-6).

**Table 4:** Awareness of participants about inguinal hernia (n=461)

		No.	%
Definition of inguinal hernia	Incorrect answer	164	35.6%
	Correct answer	297	64.4%
The site of the inguinal hernia located	Incorrect answer	237	51.4%
	Correct answer	224	48.6%
Individuals at increased risk of inguinal hernia	Incorrect answer	140	30.4%
	Correct answer	321	69.6%
Patients most likely to develop inguinal hernia	Incorrect answer	327	70.9%
	Correct answer	134	29.1%
Symptoms of inguinal hernia	Incorrect answer	0	0.0%
	Correct answer	461	100.0%
Awareness about the diagnosis of inguinal hernia	Incorrect answer	180	39.0%
	Correct answer	281	61.0%
Awareness about the treatment of inguinal hernia	Incorrect answer	321	69.6%
	Correct answer	140	30.4%
Awareness about the prevention of inguinal hernia	Incorrect answer	130	28.2%
	Correct answer	331	71.8%

Table 5 indicates that the level of awareness was significantly lower among the 18-25 years age group (median:) than the older age groups (median: 5 and 6, respectively; p<0.001). In addition, the median awareness score was significantly lower among undergraduates than graduates (median=5 and 6, respectively; p<0.001). Alternatively, neither sex nor place of residence were significantly associated with awareness (p=0.1444 and 0.267, respectively).

**Table 5:** Comparison of awareness and the sociodemographic characteristic * Significant at p<0.05; IQR: interquartile range.

Social factor		Awareness score		p-Value
		Median	IQR	
Sex	Female	5.0	4.0–6.0	0.144
	Male	5.0	2.0–6.0	
Age groups, year	18–25	5.0	2.0–6.0	<0.001*
	2–-33	6.0	4.0–7.0	
	34–45	6.0	5.0–7.0	
	4–-59	6.0	5.0–7.0	
Education	Below university	5.0	2.0–6.0	<0.001*
	University	6.0	4.0–7.0	
Residence	Rural	5.0	2.06.0	0.267
	Urban	5.0	3.0–7.0	

## Discussion

Inguinal hernia is a common type of hernia, which can lead to various outcomes, which includes life-threatening complications such as strangulation. Therefore, this study aimed to evaluate the prevalence, risk factors, and awareness of inguinal hernias among adults in Saudi Arabia. Our findings indicate that the prevalence of inguinal hernia was 5.2%. Significant risk factors for inguinal hernia include a family history of inguinal hernia, chronic cough, bronchial asthma, and smoking. Awareness levels were significantly lower among undergraduates and in the 18-25 age group.

Most study participants were unmarried men aged between 18 and 25 years, with a bachelor's degree and a yearly income of less than 50,000 Saudi Riyals. They resided primarily with their parents in urban areas. These characteristics are consistent with those of several previous studies conducted worldwide and in Saudi Arabia [13,16-22].

Alqahtani et al. reported a higher prevalence of inguinal hernias (12.3%) in an athletic population in Saudi Arabia [22]. Furthermore, the prevalence of inguinal hernia was high (27.3%) in the Arar region [16]. The several international estimates included 70.2% in Nigeria [23], 56% in Egypt [15], 21.8% in India [24], 29.8% in Ethiopia [25], 20.9% in Brazil [26], 13% in the Ashanti region of Ghana [27], and 9.4% in Uganda [28]. The discrepancy in the prevalence of inguinal hernia between these studies may be due to the differences in population characteristics.

Our study identified associations between inguinal hernias and several common risk factors, including family history, chronic cough or bronchial asthma, smoking, heavy lifting, and chronic constipation. Multivariate regression analysis revealed that family history of hernia, chronic cough or asthma, and smoking were significant risk factors for inguinal hernia.

We discovered that 45.8% of participants with inguinal hernias reported a positive family history. Khalaf A [19] stated that 20.8% of their participants had a family history of inguinal hernia. Likewise, Lau et al. [13] and Junge et al. [29] emphasized the predictive significance of a positive family history of inguinal hernias. Alqahtani et al. discovered that strenuous work activities and a history of hernia were significant predictors of inguinal hernia in Saudi athletes [22]. Genetic predisposition increases the likelihood of inguinal hernia. The risk of hernia development is high if a first-degree relative has this condition [30].

Increased intra-abdominal pressure due to excessive coughing and straining in patients with asthma may contribute significantly to the development of inguinal hernias. Asthma is a well-established risk factor for hernias [31], as confirmed by numerous studies conducted in Saudi Arabia [14,32]. An earlier study reported that a high total activity index and a positive family history were risk factors for both types of inguinal hernia [13]. However, only a direct inguinal hernia was significantly associated with chronic obstructive pulmonary disease.

Hernia development may be linked to collagenous tissues affected by harmful factors such as smoking, alcohol consumption, and diabetes. Smoking significantly increases the risk of inguinal hernia recurrence. This may be attributed to its interference with the standard metabolism of the connective tissue [33]. However, quantifiable results are necessary to accurately demonstrate tissue changes, particularly in cremaster muscle [34].

Heavy lifting was significantly associated with inguinal hernia in the present study. The debate regarding the connection between strenuous lifting and the development of hernias is ongoing. Flitch et al. revealed a significant association between physical exertion and the incidence of inguinal hernia [12]. Individuals engaged in jobs involving lifting or physically demanding activities were at a higher risk than those with jobs that demanded fewer activities. However, a systematic review by Svendsen et al., 2013 [35] has provided inconclusive evidence regarding the association between frequent or occasional heavy lifting or a single strenuous lifting event and the onset of groin hernia. Weightlifters did not demonstrate a higher incidence of inguinal hernias. However, family history and history of hernia were considered for weightlifters [10].

Specific actions and medical conditions have been reported to increase the pressure on the abdominal wall, such as prolonged straining during constipation, which may result in the development of an inguinal hernia. However, no significant correlation was found between an enlarged prostate, straining during urination, and inguinal hernia. This could be due to the small sample size of this study [21].

In the present study, the prevalence of inguinal hernias was not significantly correlated with sex. Nonetheless, men presented with a slightly higher prevalence of hernias than women. Similarly, Alsaigh et al. found no statistical correlation between sex and hernia prevalence [36]. However, these results contradict the findings of Alqahtani et al., who reported a significant correlation between male athletes and inguinal hernia [22], and AhmedAlenazi et al., who found a higher prevalence in women than men [17].

The median awareness score of our participants regarding inguinal hernias was 5 out of 8. Alsaigh et al. observed that adults in Saudi Arabia have a moderate-level knowledge of hernia [36]. According to Bahareth et al., over 50% of their study participants demonstrated a moderate understanding of hernias, including their risk factors and complications [37]. However, Albukairi et al. discovered an insufficient public awareness of the factors that make young Saudis in Riyadh more prone to hernias [14]. The study revealed that merely 48% of the participants established a link between hernias and the primary underlying risk factors, 22% of the respondents denied any correlation, and 30% admitted not knowing about the risk factors and their connection to the development of hernias. Alkalash et al. identified a lack of understanding of hernias [20]. A correlation was found between possessing knowledge of hernias and being young, unmarried, or a student.

In this study, the awareness level was significantly lower among the 18-25 age group than in the older age groups, and the individuals with a below university education displayed notably low awareness levels. Individuals with college degrees have a better understanding of inguinal hernias and their associated risk factors than those with less education [14,15,33,35,38]. This study indicated that individuals between the ages of 22 and 28 years possessed more knowledge about abdominal hernias than other age groups, which is attributed to the higher proportion of recent graduates in this age range. It is vital to raise awareness among those under the age of 25 years, as they appear to lack a fundamental understanding of hernias and their associated risks. Contrary to our findings, Mahfouz et al. revealed that 18- to 24-year-olds had a greater understanding of inguinal hernia than those of the other age groups [18]. The difference in knowledge levels could be due to the variations in the assessment methods and questionnaire design.

Acquiring precise knowledge of risk factors and other aggravating factors helps patients and caregivers effectively manage diseases, reduce associated consequences, and enhance quality of life. Healthcare professionals, media, and the Ministry of Health are responsible for disseminating information and educating the public regarding the various variables that contribute to the development of abdominal hernias.

Limitations

First, this cross-sectional study had a limited sample size, which hindered the establishment of cause-and-effect relationships. Second, there is a possibility of personal bias in self-reported questionnaires, leading to overestimation or underestimation of the condition. Therefore, it may be difficult to generalize these findings to the entire Saudi population. Further investigations using larger sample sizes based on clinical diagnoses and hospital data are required to obtain a more precise estimate of hernia prevalence.

## Conclusions

The prevalence of inguinal hernia among adult individuals in Saudi Arabia is low (5.2%). An association was found between inguinal hernia and a family history of chronic cough or bronchial asthma, smoking, heavy lifting, and constipation. Family history of hernia, chronic cough or asthma, and smoking were identified as significant risk factors for inguinal hernia. Overall awareness was moderate, with lower levels among students and those in the 18-25 years age group.
